# COVID-19: Current understanding of its Pathophysiology, Clinical presentation and Treatment

**DOI:** 10.1136/postgradmedj-2020-138577

**Published:** 2020-09-25

**Authors:** Anant Parasher

**Affiliations:** Medicine, Guru Teg Bahadur Hospital, New Delhi, India

**Keywords:** Virology

## Abstract

**Background:**

The severe acute respiratory syndrome (SARS) coronavirus-2 is a novel coronavirus belonging to the family Coronaviridae and is now known to be responsible for the outbreak of a series of recent acute atypical respiratory infections originating in Wuhan, China. The disease caused by this virus, termed coronavirus disease 19 or simply COVID-19, has rapidly spread throughout the world at an alarming pace and has been declared a pandemic by the WHO on March 11, 2020. In this review, an update on the pathophysiology, clinical presentation and the most recent management strategies for COVID-19 has been described.

**Materials and Methods:**

A search was conducted for literature and various articles/case reports from 1997 to 2020 in PUBMED/MEDLINE for the keywords coronavirus, SARS, Middle East respiratory syndrome and mRNA virus.

**Results and Conclusions:**

COVID-19 has now spread globally with increasing morbidity and mortality among all populations. In the absence of a proper and effective antibody test, the diagnosis is presently based on a reverse-transcription PCR of nasopharyngeal and oropharyngeal swab samples. The clinical spectrum of the disease presents in the form of a mild, moderate or severe illness. Most patients are either asymptomatic carriers who despite being without symptoms have the potential to be infectious to others coming in close contact, or have a mild influenza-like illness which cannot be differentiated from a simple upper respiratory tract infection. Moderate and severe cases require hospitalisation as well as intensive therapy which includes non-invasive as well as invasive ventilation, along with antipyretics, antivirals, antibiotics and steroids. Complicated cases may require treatment by immunomodulatory drugs and plasma exchange therapy. The search for an effective vaccine for COVID-19 is presently in full swing, with pharmaceutical corporations having started human trials in many countries.

## INTRODUCTION

A series of acute atypical respiratory infections ravaged the Wuhan city of Hubei province of China in December 2019. The pathogen responsible for these atypical infections was soon discovered to be a novel coronavirus belonging to the family Coronaviridae and was named as the severe acute respiratory syndrome coronavirus-2 (SARS-CoV-2). It was seen to be highly homologous to the SARS coronavirus (SARS-CoV), which was responsible for the respiratory pandemic during the 2002–2003 period.^1  2^ The respiratory illness caused by this virus was termed as coronavirus disease 2019 or simply COVID-19 by the WHO, and the outbreak was considered to have started via a zoonotic spread from the seafood markets in Wuhan, China. Subsequently, human-to-human transmission was recognised to be responsible for the community spread of the disease, being reported in approximately 200 countries worldwide.^3, 4, 5, 6^

After being broadcast as a public health emergency on January 30, 2020, COVID-19 was subsequently declared a pandemic on March 11, 2020 by the WHO. The SARS-CoV-2, which initially led to a severe pneumonia outbreak in China, has now rapidly spread all throughout the globe. As of July 6, 2020, there were almost 11.5 million cases worldwide, with approximately 536 893 reported deaths.^7  8^

## VIRAL LIFE CYCLE AND HOST CELL INVASION

The virus is transmitted via respiratory droplets and aerosols from person to person. Once inside the body, the virus binds to host receptors and enters host cells through endocytosis or membrane fusion. The coronaviruses are made up of four structural proteins, namely, the spike (S), membrane (M), envelop (E) and nucleocapsid (N) proteins.^6  9^ The S protein is seen to be protruding from the viral surface and is the most important one for host attachment and penetration. This protein is composed of two functional subunits (S_1_ and S_2_), among which S_1_ is responsible for binding to the host cell receptor and S_2_ subunit plays a role in the fusion of viral and host cellular membranes.^6^

ACE-2 has been identified as a functional receptor for SARS-CoV and is highly expressed on the pulmonary epithelial cells.^10^ It is through this host receptor that the S protein binds initially to start the host cell invasion by the virus.^11, 12, 13^ After binding of SARS-CoV-2 to the ACE-2, the S protein undergoes activation via a two-step protease cleavage: the first one for priming at the S1/S2 cleavage site and the second cleavage for activation at a position adjacent to a fusion peptide within the S_2_ subunit.^14, 15, 16, 17^ The initial cleavage stabilises the S2 subunit at the attachment site and the subsequent cleavage presumably activates the S protein causing conformational changes leading to viral and host cell membrane fusion.^18^

**Figure 1:**
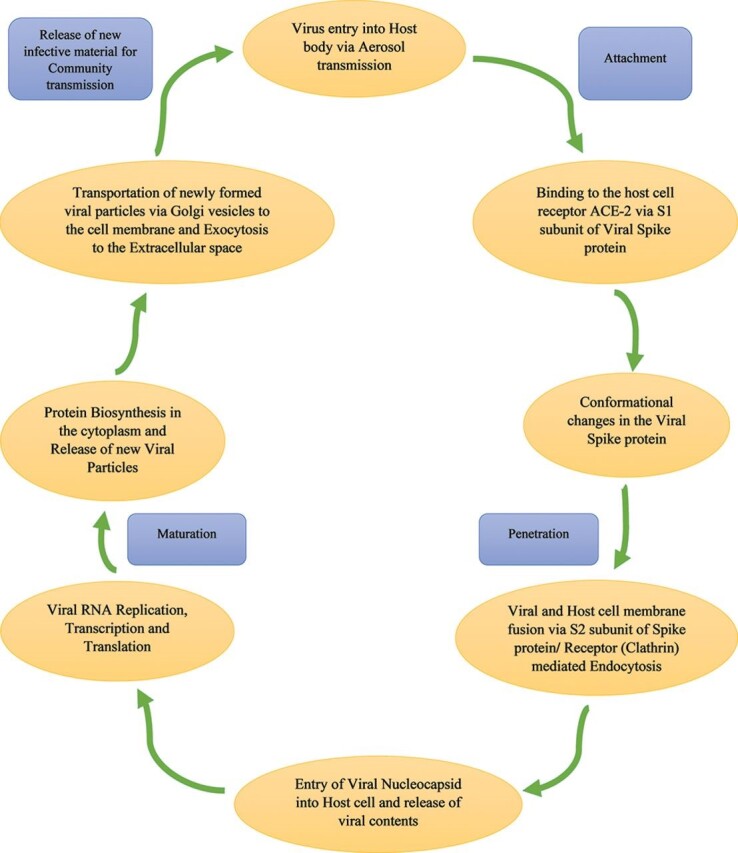
The severe acute respiratory syndrome coronavirus-2 life cycle.

Postmembrane fusion, the virus enters the pulmonary alveolar epithelial cells and the viral contents are released inside. Now inside the host cell, the virus undergoes replication and formation of a negative strand RNA by the pre-existing single-strand positive RNA through RNA polymerase activity (transcription). This newly formed negative strand RNA serves to produce new strands of positive RNAs which then go on to synthesise new proteins in the cell cytoplasm (translation).^19, 20, 21^ The viral N protein binds the new genomic RNA and the M protein facilitates integration to the cellular endoplasmic reticulum. These newly formed Nucleocapsids are then enclosed in the ER membrane and transported to the lumen, from where they are transported via golgi vesicles to the cell membrane and then via exocytosis to the extracellular space. The new viral particles are now ready to invade the adjacent epithelial cells as well as for providing fresh infective material for community transmission via respiratory droplets.^6^ An overview of the viral life cycle is shown in figure 1.

## DISEASE PATHOPHYSIOLOGY

**Figure 2:**
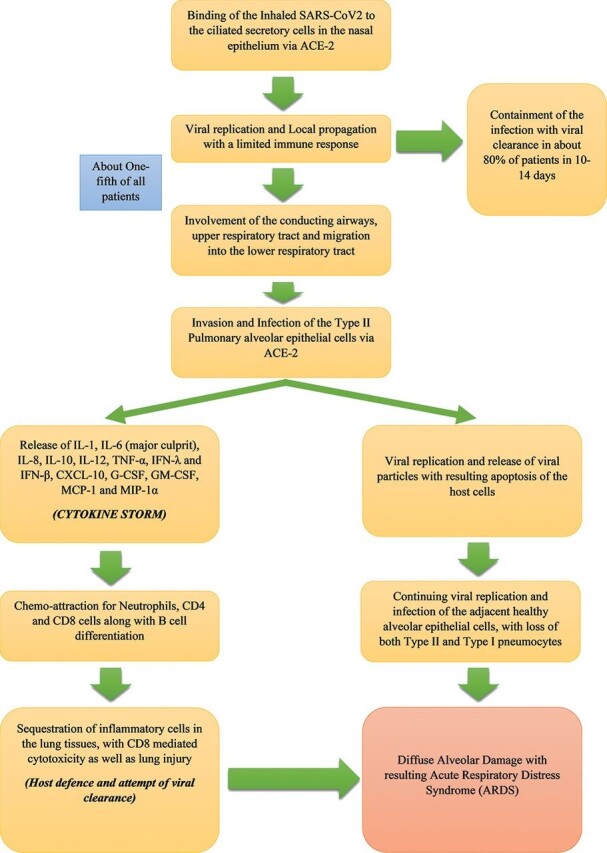
Pathophysiology of COVID-19. CXCL-10, C-X-C motif chemokine ligand 10; IFN, interferon; IL, interleukin; MCP-1, monocyte chemoattractant protein-1; MIP-1α, macrophage inflammatory protein-1α; SARS-CoV-2, severe acute respiratory syndrome coronavirus-2; TNF-α, tumour necrosis factor-α; G-CSF, granulocyte colony-stimulating factor; GM-CSF, granulocyte-macrophage colony-stimulating factor.

Although much has been discovered regarding the transmission and presentation, less is known about the pathophysiology of COVID-19. An overview of the disease pathophysiology has been shown in figure 2.^6  22  23^

## ASYMPTOMATIC PHASE

The SARS-CoV-2 which is received via respiratory aerosols binds to the nasal epithelial cells in the upper respiratory tract. The main host receptor for viral entry into cells is the ACE-2, which is seen to be highly expressed in adult nasal epithelial cells.^24  25^ The virus undergoes local replication and propagation, along with the infection of ciliated cells in the conducting airways.^26^ This stage lasts a couple of days and the immune response generated during this phase is a limited one. In spite of having a low viral load at this time, the individuals are highly infectious, and the virus can be detected via nasal swab testing.

## INVASION AND INFECTION OF THE UPPER RESPIRATORY TRACT

In this stage, there is migration of the virus from the nasal epithelium to the upper respiratory tract via the conducting airways. Due to the involvement of the upper airways, the disease manifests with symptoms of fever, malaise and dry cough. There is a greater immune response during this phase involving the release of C-X-C motif chemokine ligand 10 (CXCL-10) and interferons (IFN-β and IFN-λ) from the virus-infected cells.^27^ The majority of patients do not progress beyond this phase as the mounted immune response is sufficient to contain the spread of infection.

## INVOLVEMENT OF THE LOWER RESPIRATORY TRACT AND PROGRESSION TO Acute RESPIRATORY DISTRESS SYNDROME (ARDS)

About one-fifth of all infected patients progress to this stage of disease and develop severe symptoms. The virus invades and enters the type 2 alveolar epithelial cells via the host receptor ACE-2 and starts to undergo replication to produce more viral Nucleocapsids. The virus-laden pneumocytes now release many different cytokines and inflammatory markers such as interleukins (IL-1, IL-6, IL-8, IL-120 and IL-12), tumour necrosis factor-α (TNF-α), IFN-λ and IFN-β, CXCL-10, monocyte chemoattractant protein-1 (MCP-1) and macrophage inflammatory protein-1α (MIP-1α). This ‘cytokine storm’ acts as a chemoattractant for neutrophils, CD4 helper T cells and CD8 cytotoxic T cells, which then begin to get sequestered in the lung tissue. These cells are responsible for fighting off the virus, but in doing so are responsible for the subsequent inflammation and lung injury. The host cell undergoes apoptosis with the release of new viral particles, which then infect the adjacent type 2 alveolar epithelial cells in the same manner. Due to the persistent injury caused by the sequestered inflammatory cells and viral replication leading to loss of both type 1 and type 2 pneumocytes, there is diffuse alveolar damage eventually culminating in an acute respiratory distress syndrome.^6  28^

## VIRAL TRANSMISSION AND CLINICAL FEATURES

COVID-19 virus is mainly spread from person to person via respiratory droplet transmission, which occurs when a person is in close contact with someone who is actively coughing or sneezing. This occurs through exposure of the mucosal surfaces of the host, that is, eyes, nose and mouth, to the incoming infective respiratory droplets.^3 29–33^ Transmission of the virus may also occur through fomites used by or used on the infected individual such as bedsheets, blankets, kitchen utensils, thermometers and stethoscopes. Airborne transmission has not been reported for COVID-19, except in specific circumstances in which procedures that generate aerosols are performed, that is, endotracheal intubation, bronchoscopy, open suctioning, nebulisation with oxygen, bronchodilators or steroids, bag and mask ventilation before intubation, tracheostomy and cardiopulmonary resuscitation.^29  33  34^

The incubation period of COVID-19, which is the time period from exposure to the virus to symptom onset, is 5–6 days, but can be up to 14 days. During this period, also known as the ‘pre-symptomatic’ period, the infected individuals can be contagious and transmit the virus to healthy individuals in the population.^35^ The patients of COVID-19 belong mostly to the 40–70 years age group, and most commonly present with fever, body aches, breathlessness, malaise and dry cough, although patients may present with asymptomatic, mild, moderate or severe disease (table 1).^1 36–39^ Some patients may also present with gastrointestinal symptoms such as abdominal pain, vomiting and loose stools.^40  41^ The complications seen in patients with COVID-19 infection are caused mostly due to the ‘cytokine storm’ and are summarised in table 2.^42^

**Table 1:** Clinical spectrum of COVID-19 disease^1 36–41^

Severity of disease	Presentation
Asymptomatic	No clinical symptoms Positive nasal swab test Normal chest X-ray
Mild illness	Fever, sore throat, dry cough, malaise and body aches or Nausea, vomiting, abdominal pain, loose stools
Moderate illness	Symptoms of pneumonia (persistent fever and cough) without hypoxemia Significant lesions on high-resolution CT chest
Severe illness	Pneumonia with hypoxemia (SpO_2_ < 92%)
Critical state	Acute respiratory distress syndrome, along with shock, coagulation defects, encephalopathy, heart failure and acute kidney injury

**Table 2:** Complications seen in patients with COVID-19^42^

Frequency	Complication
Commonly seen	Acute respiratory distress syndrome Acute respiratory failure Sepsis Disseminated intravascular coagulation Acute liver and kidney injury Pulmonary embolism
Rare	Rhabdomyolysis Multisystem inflammatory syndrome Aspergillosis Pancreatitis Autoimmune haemolytic anaemia Neurological complications

## DIAGNOSIS AND IMAGING

### Molecular tests (RT-PCR)

Samples are collected from the upper respiratory tract via nasopharyngeal and oropharyngeal swabs and from the lower respiratory tract via expectorated sputum and bronchoalveolar lavage (only for mechanically ventilated patients). After being stored at 4°C, the samples are sent to the laboratory where amplification of the viral genetic material is done through a reverse-transcription process.^6^ This involves the synthesis of a double-stranded DNA molecule from the existing viral RNA by either reverse-transcription PCR (RT-PCR) or a real-time RT-PCR.^43  44^ Finally, the conserved portions of the SARS-CoV-2 genetic code are identified on the amplified genetic material.^6^

The test is recommended to be repeated for verification in cases of a positive test and again to confirm viral clearance in COVID-19 positive cases. The sensitivity of these tests is not very high, that is, approximately 53.3% of COVID-19-confirmed patients had positive oropharyngeal swabs, and about 71% of patients came out to be RT-PCR positive with sputum samples.^45  46^ The RT-PCR results usually show positivity after 2–8 days.^47^

### Serology

Till date, no effective antibody test has been developed. A centers for disease control and prevention (CDC) research on a test developed by the US Vaccine Research Centre at the National Institutes of Health is ongoing, which seems to have a specificity higher than 99% with a sensitivity of 96%.^6^

### Blood tests

- A normal or decreased white blood cell count (and lymphopenia) can be observed in many cases, which is also considered to be indicative of a worse prognosis.
- Increased levels of lactate dehydrogenase, C reactive protein, creatine kinase (CK MB and CK MM), aspartate amino-transferase and alanine amino-transferase can be seen.^6^
- Increased D-dimer levels and an elevated neutrophil-to-lymphocyte ratio are seen in some patients.^48^
- Coagulation abnormalities can be observed in severe cases, as indicated by increase in prothrombin time and international normalised ratio.

### Chest X-ray

Chest X-ray is usually inconclusive in the early stages of the disease and might not show any significant changes. As the infection progresses, bilateral multifocal alveolar opacities are observed, which may also be associated with pleural effusion.^6^

### CT

High-resolution CT (HRCT) is extremely sensitive and the method of choice for diagnosing COVID-19 pneumonia, even in initial stages of the illness. The most commonly seen features are multifocal bilateral ‘ground-glass’ areas associated with consolidation and a patchy peripheral distribution, with greater involvement of the lower lobes. A ‘reversed halo sign’ is also seen in some patients, which is identified as a focal area of patchy opacities surrounded by a peripheral ring with consolidation. Other findings include pleural effusion, cavitation, calcification, and lymphadenopathy.^6^

A brief summary of all investigations designed for COVID-19 is given in table 3.

**Table 3:** Investigations for COVID-19

Investigation	Remarks
Basic blood work	Decreased WBC count as well as lymphopenia Increased levels of AST and ALT, LDH and CRP Increased D-dimer Increased PT/INR
Molecular testing via RT-PCR	Techniques employed are RT-PCR and rRT-PCR which amplify viral genetic material obtained via nasal swab Poor sensitivity Repeat testing required for verification of viral clearance
Chest X-ray	No significant findings early in the disease Bilateral patchy opacities in advanced disease
HRCT chest	Multifocal bilateral ‘ground or ground-glass’ areas associated with consolidation areas with patchy distribution ‘Reverse halo’ sign Cavitation, calcification and lymphadenopathy High sensitivity for COVID-19 diagnosis
Serology/antibody testing	Further research still required for a proper/sensitive antibody test

ALT, alanine amino-transferase; AST, aspartate amino-transferase; CRP, C reactive protein; HRCT, high-resolution CT; INR, international randomised ratio; LDH, lactate dehydrogenase; PT, prothrombin time; RT-PCR, reverse-transcription PCR; rRT-PCR, real-time reverse-transcriptionPCR; WBC, white blood count.

## MANAGEMENT STRATEGIES

**Figure 3:**
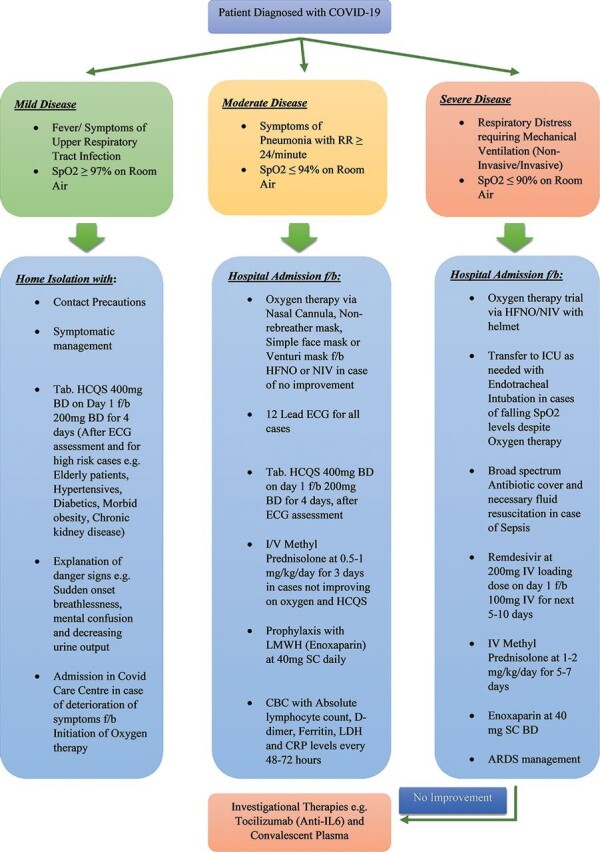
Treatment protocol for patients with COVID-19. RR, respiratory rate; SpO2, oxygen saturation; HCQS, hydroxychloroquine; ECG, electrocardiogram; HFNO, high flow nasal oxygen; NIV, non-invasive ventilation; I/V, intravenous; LMWH, low molecular weight heparin; CBC, complete blood count; LDH, lactate dehydrogenase; CRP, c- reactive protein; ICU, intensive care unit; ARDS, acute respiratory distress syndrome.

As no vaccine is presently available for COVID-19, the treatment is mainly symptomatic and supportive in most cases. Initially, the patient presenting to the emergency is categorised into mild, moderate or severe according to the symptoms on presentation. Most patients present with mild-to-moderate symptoms such as fever, persistent dry cough, body aches and occasional breathlessness. A small fraction of patients may also present with acute respiratory failure and acute respiratory distress syndrome with associated sepsis or multiorgan failure. The complete management protocol for patients with COVID-19 is depicted in figure 3.

### Mild cases (SpO_2_ levels of 94%–97% in room air)

#### Oxygen therapy via nasal cannula/simple face mask/venturi mask/non-rebreather mask

For management of patients presenting with respiratory distress due to COVID-19, the emergency room should be well-stocked with functioning oxygen systems, pulse oximeters and single-use disposable oxygen-delivering interfaces such as nasal cannulas, simple face masks, venturi masks, non-rebreather (NRB) masks and masks with reservoir bag.^49^

Oxygen therapy is started with the arrival of the patient in the emergency and is administered according to the severity of presentation. For patients presenting with mild breathlessness and a SpO_2_ level between 94% and 97%, a simple face mask or a nasal cannula can be used for oxygen delivery. In patients maintaining a SpO_2_<94%, patients with chronic obstructive pulmonary disease (COPD), a respiratory rate >30/min or persistent dyspnoea, oxygen is to be administered via a 40% venturi mask to ensure a higher level of fixed oxygen delivery. Reassessment is to be done after 10 min and if stable again at 6 hours.^6^ If there is little or no improvement after 6 hours on a venturi mask, non-invasive ventilation (NIV) is to be considered.^6^ NRB masks limit the dispersion of aerosol, thereby offering a safer alternative for supplemental oxygen delivery.^50^

In addition to oxygen therapy, mild cases may be managed on a symptomatic basis with antipyretics (acetaminophen) for fever and pain, oral fluid supplementation and adequate nutrition. Hydroxychloroquine (HCQS) may be considered for cases having high-risk features such as age greater than 60 years and comorbidities such as morbid obesity, hypertension, COPD, diabetes, chronic kidney/liver disease and cerebrovascular disease.^49^

### Moderate cases (SpO_2_ levels of 90%–94% in room air)

Patients with moderate disease (SpO_2_ of 90%–94% in room air) are to be isolated to contain the virus transmission. A detailed clinical history is to be taken including history of pre-existing comorbid conditions. There should be monitoring of vital signs and oxygen saturation (SpO_2_ levels), along with investigations such as a complete blood count, ECG and chest X-ray examination.^49  51^

### High-flow nasal oxygen (HFNO) therapy and NIV

HFNO therapy is used in these cases where it is not possible to maintain SpO_2_>92% and/or there is no improvement in dyspnoea through standard oxygen therapy via face mask. The oxygen flow rate in HFNO therapy is roughly 30–40 L/min, and it is to be continuously adjusted according to the clinical response of the patient. It is also found to be beneficial for continuous positive airway pressure (CPAP) breaks between cycles as well as in critically ill patients for whom assisted fibre-optic tracheal intubation is required.^52^ This therapy should not be used in a hypercapnic patient, and owing to a higher risk of aerosolisation, it must only be used in negative pressure rooms. Patients who do not improve after an hour with flow >50 L/min and FiO2>70% are recommended to be switched over to NIV.

NIV by CPAP has an important role in managing the respiratory failure caused due to COVID-19. NIV is usually administered through a full face mask or an oro-nasal mask, but can also be given via a helmet in order to reduce aerosolisation. CPAP is started with 8–10 cm H_2_O and FiO_2_ 60% and adjusted according to patient compliance.^6^

Other therapies in moderate COVID-19 disease include HCQS 400 mg two times per day stat followed by 200 mg two times per day for next 4 days (in cases without evidence of cardiac disease), intravenous methylprednisolone 0.5–1 mg/kg for 3 days (preferably within 48 hours of admission), and anticoagulation via prophylactic dose of low molecular weight heparin (LMWH) (enoxaparin 40 mg per day sub-cutaneous (SC)).^6  49  53^Antibiotics are recommended for management of secondary bacterial infections. The patient is to be monitored for signs of haemodynamic instability and increased oxygen demand as indicated by the use of accessory muscles of respiration.

Although there have been concerns regarding aerosol generation with the use of HFNO therapy and NIV, negative pressure rooms and administration of oxygen through a well-fitting helmet, respectively, have largely addressed this issue. Patients receiving HFNO therapy should be monitored by personnel who have experience with endotracheal intubation in case the patient does not improve after a short duration or decompensates abruptly.

### Severe cases (SpO_2_ levels ≤90% in room air or patients with ARDS)

For patients presenting with severe disease/ARDS, immediate oxygen therapy is to be started at 5 L/min and titrate flow rate for a target of SpO_2_≥90% in non-pregnant adults and SpO_2_≥92–96% in pregnant patients.^49^ As compared to standard oxygen therapy delivered via face mask, HFNO therapy is much more effective in reducing the need for intubation in these cases. In cases of hypercapnia (exacerbation of obstructive lung disease and cardiogenic pulmonary oedema), haemodynamic instability, multiorgan failure, abnormal mental status or worsening of oxygen saturation below 90%, invasive ventilation via endotracheal intubation has to be considered promptly.^49  51^

### Endotracheal intubation and mechanical ventilation

Endotracheal intubation is usually performed by specialised personnel, after donning all personal protective equipment such as full-body gown, a N95 mask and protective goggles. Preoxygenation with 100% oxygen for 5 min is done via the CPAP method, and if possible, rapid sequence intubation should be preferred. Mechanical ventilation is initiated with lower tidal volumes (4–8 mL/kg body weight) and lower inspiratory pressures (plateau pressure <30 cm H_2_O). In patients with severe ARDS, prone ventilation for 16–18 hours per day is recommended but requires sufficient human resources and expertise to be performed safely. In patients with moderate or severe ARDS, higher positive end-expiratory pressure (PEEP) is suggested which has the benefits of decreasing trauma due to atelectasis and increased recruitment of alveoli, but can cause complications due to lung over-distension and increase in the pulmonary vascular resistance.^49  51^

Extracorporeal membrane oxygenation (ECMO) for patients with refractory hypoxemia despite endotracheal intubation and mechanical ventilation should be considered if feasible. In COVID-19 patients, ECMO may represent an efficient support in case of refractory hypoxemia and/or cardiogenic/septic shock unresponsive to maximal therapy.^54^

## MANAGEMENT OF SEPSIS/SEPTIC SHOCK

Septic shock in adults can be confirmed when, along with the background of a definite infection, there is presence of tachycardia, tachypnoea, hyperthermia, increased lactate levels and the requirement of vasopressor support to maintain mean arterial pressure ≥65 mm Hg, all in the absence of hypovolemia.^49^ Management includes broad-spectrum antibiotics, fluid resuscitation and vasopressors for prevention/management of shock and peripheral hypo-perfusion. The preferred fluids in cases of septic shock are usually isotonic crystalloids (normal saline and ringer’s lactate), given at the rate of at least 30 mL/kg in the first 3 hours. Excess fluid resuscitation may lead to signs of volume overload (raised jugular venous pressure, chest crepitations and hepatomegaly) and requires discontinuation or reduction of intravenous fluids. Dobutamine is to be started if the patient shows signs of poor perfusion and cardiogenic shock despite the ongoing antibiotic and vasopressor support.^49  51^

## OTHER THERAPIES FOR COVID-19

### Antibiotics

Although not always recommended in viral pneumonia, an optimum and effective antibiotic regimen helps prevent or manage secondary bacterial infections and sepsis. Macrolides such as azithromycin are quite effective in preventing pulmonary infections in patients with viral pneumonias, in addition to having a significant anti-inflammatory effect on the airways.^55^

### Corticosteroids

Steroids can be used for a short period of time, that is, 3–5 days in patients who show progressive deterioration of oxygen saturation, increased activation of the pro-inflammatory response and rapid worsening of features on chest imaging. Methylprednisolone was the first and only steroid indicated initially, at a dose not exceeding 0.5–1 mg/kg/day for moderate cases and 1–2 mg/kg/day for severe cases. Higher doses were not recommended in view of the delay in viral clearance due to steroid mediated immunosuppression.^32  49  56  57^

Recently, dexamethasone has also been found to be effective for decreasing mortality in severe and critically ill cases.^58^

### Antiviral drugs

The following antiviral drugs have been put to use for COVID-19 patients so far.

#### Remdesivir (CIPREMI/COVIFOR)

It was initially suggested by some preclinical studies that remdesivir has in vitro activity against multiple RNA viruses (including Ebola) and could be beneficial for both prophylaxis and treatment of coronavirus infections.^6  49  59^ Remdesivir is a broad-spectrum antiviral agent, and acts by blocking the action of viral RNA-dependent RNA polymerase. This causes evasion of proofreading by viral exoribonuclease, causing a significantly decreased production of viral RNA.^60^ In a mouse model of SARS-CoV, remdesivir was observed to reduce the lung viral load and improve pulmonary function.^61^ It was used to treat the first case of COVID-19 infection in the USA, who showed rapid improvement after 1 day of remdesivir treatment.^62^

In two separate studies, although remdesivir was seen to be superior to placebo in decreasing rates of lower respiratory tract infections and shortening hospital stay, there was no significant difference seen between a 5-day course and a 10-day course of remdesivir.^63  64^ In comparison, therapeutic doses of lopinavir (LPV)/ritonavir (RTV) although did improve pulmonary function, but were not able to reduce virus replication or prevent severe lung damage. Thus, it is indicated that remdesivir has shown more potential than LPV/RTV in the treatment of COVID-19.^65  66^ It may be considered in patients with moderate disease at a loading dose of 200 mg intravenous over 1–2 hours on day 1, followed by 100 mg intravenous daily for 5–10 days. Contraindications to the use of remdesivir include use in children, pregnant or lactating females, and patients with severe hepatic or renal impairment.^49^

Thus, it is implied that remdesivir is best suited for hospitalised patients with COVID-19 having moderate-to-severe disease, and requiring supplemental oxygen therapy. On May 1, 2020, the US Food and Drug Administration (USFDA) gave emergency use approval for remdesivir in patients hospitalised with severe COVID-19; the final approval being given in light of tentative evidence of remdesivir efficacy in such patients. However, it is to be noted that treatment with remdesivir alone is not likely to be sufficient given the high mortality despite its use.

#### Lopinavir/ritonavir (KALETRA)

LPV has been shown to inhibit the coronavirus protease activity in vitro and in animal studies and to lower mortality rates as seen in a cohort study.^67^ Effective dose of LPV is 400 mg orally every 12 hours, and based on the effectiveness of this drug during the previous SARS and Middle East respiratory syndrome virus outbreaks, it was initially seen as a potential treatment option for COVID-19.^68^ However, a recent randomised controlled trial demonstrated no definitive benefit of LPV/RTV therapy as compared to routine management protocol.^69^

#### Oseltamivir (TAMIFLU)

Although designed and used against influenza virus outbreaks, oseltamivir (75 mg two times per day for 5 days) was tested for patients with COVID-19 along with standard supportive care in two case series from Wuhan, China. As such, no clear additional benefit of oseltamivir therapy was observed in these patients.^70  71^

#### Favipiravir (FABIFLU)

Favipiravir shows activity against RNA viruses by conversion into the ribofuranosyl triphosphate derivative by host enzymes and subsequent selective inhibition of the viral RNA-dependent RNA polymerase. It was initially discovered by the Toyama Chemical Company in Japan for therapeutic use in resistant cases of influenza. The drug has also shown effectiveness in the treatment of avian influenza and may be an alternative option for the treatment of illness caused by pathogens such as the Ebola virus and COVID-19.^72^

Favipiravir has been recently launched under the trade name ‘FabiFlu’ by Glenmark Pharmaceuticals in June 2020 for patients with mild-to-moderate COVID-19, thereby becoming the first approved oral favipiravir medication for the treatment of COVID-19 in India. The recommended dose is 1800 mg two times per day on day 1, followed by 800 mg two times per day up until day 14. Favipiravir has proven in vitro activity against SARS CoV-2 virus and shows a significant improvement in mild-to-moderate cases with COVID-19. It is associated with rapid reduction of the viral load and an early symptomatic improvement.^72  73^

Although individual antiviral drugs have proven to be somewhat effective in mild-to-moderate cases, future strategies should evaluate combination of antiviral agents, or antivirals with other therapeutic approaches, to improve patient outcomes in the critically ill COVID-19 cases.

### Immunomodulatory drugs (tocilizumab, chloroquine and hydroxychloroquine)

#### Tocilizumab

Tocilizumab is a humanised IgG1 monoclonal antibody, directed against the IL-6 receptor and commonly used in the treatment of rheumatoid arthritis, juvenile arthritis and giant cell arteritis. It may be considered in patients with moderate disease having raised inflammatory markers (IL-6) with progressively increasing oxygen demand and in mechanically ventilated patients unresponsive to therapy. The dosage is 8 mg/kg (maximum 800 mg at one time) given slowly in 100 mL normal saline (NS) over 1 hour, which can be repeated once after 12–24 hours if needed. Active tuberculosis and neutropenia are contraindications to the use of tocilizumab.^49  74^ Treatment with tocilizumab, whether administered intravenously or subcutaneously, might reduce the risk of invasive mechanical ventilation or death in patients with severe COVID-19 pneumonia.^75^

#### Chloroquine and hydroxychloroquine

Chloroquine is a widely used antimalarial drug that has been shown to have broad-spectrum antiviral activity.^76^ Chloroquine (500 mg every 12 hours) blocks the virus infection by an increase in the endosomal pH required for virus/cell fusion, as well as by preventing SARS-CoV receptor glycosylation.^77^ It has shown efficacy in reduction of exacerbation of COVID-19 pneumonia as well as accelerated viral and symptomatic clearance.^78^

HCQS (200 mg every 12 hours) is a chloroquine analogue with a better safety profile and an anti-SARS-CoV activity in vitro.^79^ HCQS was found to be more potent than chloroquine in SARS-CoV-2-infected Vero cells and also shown to be significantly associated with viral load reduction.^80  81^ Although this antiviral effect is seen to be enhanced by the macrolide azithromycin, the combined use of both drugs can lead to an increased incidence of QT interval prolongation and cardiac arrhythmias.^82^

Both chloroquine and HCQS have been observed to have immunomodulatory effects and have the capacity to suppress the massive immune response in COVID-19 (cytokine storm) induced by mediators such as IL-1, IL-6 and IL-10.^83  84^

### Plasma exchange via convalescent plasma

It was observed that the COVID-19 virus isolated from the bronchoalveolar lavage fluid of a critically ill patient could be neutralised by plasma from several convalescent patients.^85^ This therapy may be considered in patients with severe disease who do not show improvement (oxygen requirement is progressively increasing) despite use of steroids. Some important requirements for this procedure include an adequate antibody titre in the convalescent plasma, ABO compatibility and cross-matching of the donor plasma. The recipient should be closely monitored for several hours post-transfusion for any transfusion-related adverse events and its use should be avoided in patients with IgA deficiency or Ig allergies. Dose ranges from 4 to 13 mL/kg, and usually, a single dose of 200 mL is given slowly over 2 hours.^49^ To ensure high efficacy via a high antibody titre, the convalescent plasma has to be collected within 2 weeks of patient recovery from COVID-19.^86^

### Supplementary therapies

Prophylactic anticoagulation via low molecular weight heparin (LMWH) (eg, enoxaparin 40 mg SC) should be given for anticoagulation in moderate (once time per day) to severe patients (two times per day) in view of the high risk of thromboembolism. Comorbidities such as associated hypertension, hypothyroidism or diabetes should be managed accordingly. In case of pregnant females presenting with severe disease, needful consultations should be taken from obstetric, neonatal and intensive care specialists. Psychological counselling should be ensured for patients suffering from fear and anxiety in view of being diagnosed with COVID-19.^49^

The various treatment strategies for COVID-19 are summarised in table 4.

**Table 4:** Management strategies for COVID-19

Drug/treatment	Remarks
Oxygen therapy	Nasal cannulas, simple face masks, venture masks or non-rebreather masks for mild cases HFNO therapy or NIV for moderate cases Invasive ventilation via endotracheal intubation for severe cases with ARDS
Antibiotics	Given to prevent/treat secondary bacterial infections Azithromycin is preferred in view of anti-inflammatory action
Corticosteroids	I.V methylprednisolone is recommended in moderate-to-severe cases at 1–2 mg/kg/day for 3 days Recently, dexamethasone has been found to be beneficial in severe cases
Antiviral drugs	Remdesivir has shown efficacy in moderate cases Lopinavir/ritonavir have much lower efficacy Oseltamivir has shown no clear benefit Recently launched favipiravir shows some efficacy in mild-to-moderate cases
Immunomodulatory drugs (anti-interleukins and HCQS)	Tocilizumab is a IgG1 monoclonal antibody, directed against the IL-6 receptor which is seen to be beneficial in moderate-to-severe cases of COVID-19 HCQS has shown better efficacy and safety profile as compared to chloroquine
Plasma exchange	Most beneficial if plasma collected within 2 weeks of patient recovery from disease
Anticoagulation	Enoxaparin is indicated in moderate-to-severe cases to prevent venous thromboembolism

HCQS, hydroxychloroquine; HFNO, high-flow nasal oxygen; IL, interleukin; NIV, non-invasive ventilation; I.V, intravenous; ARDS, acute respiratory distress syndrome.

## SEARCH FOR A VACCINE

The S glycoprotein of the SARS-CoV-2 is the target for most vaccines under development presently.^87^ Some of the pharmaceutical companies with vaccine development under process have been described below.^88^

### Moderna

Moderna Therapeutics announced its first experimental mRNA COVID-19 vaccine (mRNA-1273) in February 2020, ready for human testing. In May, the company announced the vaccine had produced antibodies in all 45 healthy volunteers, ages from 18 to 55, in this initial clinical phase. In early May, the company received permission from the USFDA to start a phase II study of its vaccine and expects to begin a phase III clinical trial in July.

## INOVIO

At the end of April 2020, the company had enrolled 40 healthy volunteers in its phase I clinical trial and is preparing to start a phase II/III clinical trial soon.

### University of Oxford (England)

A clinical trial with more than 500 participants showed that the potential vaccine has an 80% chance for success by using a modified virus to trigger the immune system. The university has partnered with pharmaceutical company AstraZeneca, and a late-stage clinical trial is planned to be initiated by the middle of this year.

### University of Queensland (Australia)

Preclinical testing has been started in April by growing viral proteins in cell cultures.

### CanSino Biologics and Sinovac Biotech (China)

CanSino Biologics aimed to assess the safety and immunogenicity of a recombinant adenovirus type-5 (Ad5) vectored COVID-19 vaccine expressing the S glycoprotein of the SARS-CoV-2 strain. The vaccine is seen to be tolerable, with humoral responses against SARS-CoV-2 peaking at day 28 postvaccination in healthy adults, and rapid specific T cell responses noted from day 14 postvaccination. The findings definitely warrant further investigation.^89^

Sinovac Biotech’s COVID-19 vaccine candidate, dubbed Corona Vac, induced neutralising antibodies 14 days after vaccination. More than 90% of the 600 healthy volunteers in the phase 2 part of the phase 1/2 study showed the immune response.^90^

### Other pharmaceutical companies

Johnson & Johnson have announced the initiation of early-stage human clinical trials in late July. Pfizer has also teamed up with a German company to develop a vaccine; their initial clinical trial with 200 participants is already underway in April. Human testing has already been started in the USA in early May.

### ICMR, NIV AND Zydus Cadila (India)

Recently, two COVID-19 vaccine candidates—*Covaxin*, developed by Bharat Biotech in collaboration with the Indian Council of Medical Research (ICMR) and the National Institute of Virology (NIV), and *ZyCov-D* vaccine by Zydus Cadila—have been approved for phase II and phase III human clinical trials, by the Drug Controller General of India.^91^

## CONCLUSION

The COVID-19 pandemic is now an international health emergency. Transmission via close contact from person to person has rapidly amplified the spread of disease, making it even more difficult to contain its spread in the community. The patient may be completely asymptomatic with a positive swab test, may present with a mild influenza-like illness or may present with serious symptoms that require hospitalisation. There is presently no effective antibody test available for rapid diagnosis, but HRCT scans of the chest have been seen to be quite sensitive and specific. In the absence of an effective vaccine, treatment is mainly supportive with oxygen therapy, antivirals, steroids, HCQS and antibiotics. Complicated cases and cases refractory to standard therapy may require immunomodulatory drugs and plasma exchange therapy via convalescent sera from recovered patients. Advances in viral genetic sequencing and technology have certainly paved the way for the development of a vaccine for COVID-19, with many pharmaceutical corporations already having started human trials.

**Main messages**

- The COVID-19 pandemic has now spread all across the globe, causing significant morbidity and mortality.
- To date, there is still a dire need of an effective, rapid and sensitive serology test for COVID-19.
- Several new and effective treatment options are now available, including antivirals, immunomodulators, corticosteroids and plasma exchange therapy.
- The search for a potent vaccine has been initiated by many pharmaceutical institutions around the world, with many countries having started human clinical trials.

**Key references**

1. Yuki K, Fujiogi M, Koutsogiannaki S. COVID-19 pathophysiology: a review [published online ahead of print, 20 April 2020]. *Clin Immunol* 2020;215:108427. doi:10.1016/j.clim.2020.108427.
2. Cascella M, Rajnik M, Cuomo A, *et al. Features, evaluation and treatment coronavirus (COVID-19). Stat Pearls [Internet]*. Treasure Island (FL): Stat Pearls Publishing, 2020 Jan. (Last Updated 18 May 2020).
3. Available https://www.mohfw.gov.in/pdf/Clinical/Management/Protocol/for/COVID-19.pdf. (accessed 20 Jun 2020).
4. Available https://www.who.int/docs/default-source/coronaviruse/clinical-management-of-novel-cov.pdf. (accessed 20 Jun 2020).

**Current research questions**

- What are the obstacles in developing a rapid, effectivce and sensitive serology test for COVID-19?
- Can the early implementation of HFNO and NIV improve patient prognosis in moderate to severe cases?
- Are re-infections possible in individuals already once affected by the disease?
- Can herd immunity boost the fight against COVID-19?
